# Long-term efficacy of penile rehabilitation with low-intensity extracorporeal shock wave therapy for sexual and erectile function recovery following robotic-assisted radical prostatectomy: a single-cohort pilot study

**DOI:** 10.1093/sexmed/qfad023

**Published:** 2023-05-22

**Authors:** Yuki Kohada, Takashi Babasaki, Keisuke Goto, Shogo Inoue, Yoshimasa Kurimura, Ryo Tasaka, Kenshiro Takemoto, Shunsuke Miyamoto, Kohei Kobatake, Hiroyuki Kitano, Kenichiro Ikeda, Keisuke Hieda, Tetsutaro Hayashi, Nobuyuki Hinata

**Affiliations:** Department of Urology, Graduate School of Biomedical Sciences, Hiroshima University, Hiroshima, Japan; Department of Urology, Graduate School of Biomedical Sciences, Hiroshima University, Hiroshima, Japan; Department of Urology, Graduate School of Biomedical Sciences, Hiroshima University, Hiroshima, Japan; Shobara Redcross Hospital, Hiroshima 723-0013, Japan; Chuden Hospital, Hiroshima 734-8530, Japan; Department of Urology, Graduate School of Biomedical Sciences, Hiroshima University, Hiroshima, Japan; Department of Urology, Graduate School of Biomedical Sciences, Hiroshima University, Hiroshima, Japan; Department of Urology, Graduate School of Biomedical Sciences, Hiroshima University, Hiroshima, Japan; Department of Urology, Graduate School of Biomedical Sciences, Hiroshima University, Hiroshima, Japan; Department of Urology, Graduate School of Biomedical Sciences, Hiroshima University, Hiroshima, Japan; Department of Urology, Graduate School of Biomedical Sciences, Hiroshima University, Hiroshima, Japan; Department of Urology, Graduate School of Biomedical Sciences, Hiroshima University, Hiroshima, Japan; Department of Urology, Graduate School of Biomedical Sciences, Hiroshima University, Hiroshima, Japan; Department of Urology, Graduate School of Biomedical Sciences, Hiroshima University, Hiroshima, Japan

**Keywords:** prostatic neoplasm, robot-assisted surgery, quality of life, extracorporeal shockwave therapy, erectile dysfunction

## Abstract

**Background:**

The long-term efficacy of low-intensity extracorporeal shock wave therapy (LIESWT) for penile rehabilitation after robot-assisted radical prostatectomy (RARP) has not yet been reported.

**Aim:**

To assess the long-term efficacy of LIESWT for penile rehabilitation after RARP by evaluating the postoperative recovery of sexual and erectile functions following RARP.

**Methods:**

Patients who underwent RARP at our institution were categorized into 2 groups: those who received LIESWT and those who underwent penile rehabilitation with a phosphodiesterase type 5 inhibitor (PDE5i). The control group included patients who did not undergo penile rehabilitation. Potency and scores on the Expanded Prostate Cancer Index Composite for sexual function and 5-item International Index of Erectile Function (IIEF-5) were evaluated preoperatively and over 60 months after RARP.

**Outcomes:**

The LIESWT group had significantly higher postoperative sexual function and total IIEF-5 scores and potency than the control group over the long term, and its results were not inferior to those of the PDE5i group.

**Results:**

The LIESWT, PDE5i, and control groups comprised 16, 13, and 139 patients, respectively. As compared with the control group, the LIESWT group had significantly higher sexual function scores at 6, 12, and 60 months after surgery (*P* < .05) and total IIEF-5 scores at 24 and 60 months (*P* < .05). The LIESWT group also had a significantly higher potency rate than the control group at 60 months (*P* < .05). For all time points after surgery, there were no significant differences between the LIESWT and PDE5i groups in terms of sexual function and total IIEF-5 scores and potency.

**Clinical Implications:**

LIESWT may be a new option for penile rehabilitation in patients with erectile dysfunction after RARP.

**Strengths and Limitations:**

This pilot study was performed at a single center and involved relatively few patients, which may have led to selection bias. Furthermore, the selection of this study for penile rehabilitation was not made randomly but by the patient’s choice. Despite these limitations, our results provide evidence in support of LIESWT for penile rehabilitation after RARP because this is the first study to assess the long-term efficacy of LIESWT.

**Conclusion:**

LIESWT can improve sexual and erectile functions in patients with erectile dysfunction after RARP, and its efficacy can be maintained over a long period after surgery.

## Introduction

The prostate-specific antigen test provides accessible screening for prostate cancer (PC), and localized PC is widely diagnosed in men.^1^ According to data from the American Cancer Society, PC accounted for an estimated 268 490 of all diagnosed cancer cases (27%) in males in the United States during 2021.^2^ Radical prostatectomy (RP) is the standard treatment of localized PC, and robot-assisted RP (RARP) is widely performed globally.^3^ In patients who had RP during 2017, 6560 (85.1%) in England and 4901 (68.8%) in the United States had RARP.^4^ Because of the satisfactory cancer control facilitated by RARP, postoperative quality of life has become essential. Erectile dysfunction (ED) is the most common adverse event of RARP, ranging from 10% to 46%.^5^^,^^6^ Several patients with high sexual interests have impaired postoperative quality of life due to postoperative ED.^7^

Various surgical techniques and perioperative treatments have been attempted to prevent ED after RARP. Nerve-sparing techniques are crucial for improving the sexual and erectile functions of patients who undergo RARP; however, sexual and erectile functions are rarely maintained preoperatively.^8^ Yet, penile rehabilitation involves the use of any drug or device during or after RP to maximize sexual and erectile function recovery. The most common drugs and devices for penile rehabilitation after RP are oral phosphodiesterase type 5 inhibitors (PDE5is), vacuum erection devices, intracorporeal injection therapy, and their combinations.^9^ These have been reported to improve sexual and erectile functions during treatment, but their efficacies for penile rehabilitation have not been established.^5^ Therefore, a novel method of penile rehabilitation is needed to improve postoperative ED.

Low-intensity extracorporeal shock wave therapy (LIESWT) has proven effective for various medical ischemic disorders.^10^ Recent studies have shown promising results of LIESWT in patients with vasculogenic ED.^11^ Several studies have also shown the impact of LIESWT for patients experiencing ED after RARP in improving sexual and erectile functions.^12-14^ LIESWT may become the choice of penile rehabilitation for patients with ED after RARP; however, many issues remain to be resolved.^15^ One major problem is that the long-term efficacy of LIESWT for penile rehabilitation after RARP has not been reported.

This study aimed to assess the long-term efficacy of LIESWT for penile rehabilitation after RARP. We compared the serial changes in sexual and erectile functions in the LIESWT, PDE5i, and control groups and evaluated the long-term efficacy of LIESWT for sexual and erectile function recovery following RARP.

## Methods

### Patients and study design

This pilot study retrospectively reviewed the long-term efficacy of LIESWT for penile rehabilitation after RARP. This study was approved by the Institutional Review Board of Hiroshima University Hospital (No. C-87), and informed consent was obtained from all patients. This study was carried out in accordance with the code of ethics of the World Medical Association. Patients who underwent RARP at Hiroshima University between June 2012 and June 2017 were enrolled in this study. Anterograde RARP was performed via the transperitoneal approach by the same surgeon for all patients.^16^ Given the cancer risk of each patient, we performed nerve-sparing procedures.^17^

The exclusion criteria were as follows: an unstable psychiatric or medical condition, a history of neurologic pathology, and age >75 years. In addition, patients with no preoperative potency were excluded. Potency was estimated with a question in the sexual function (SF) domain of the Expanded Prostate Cancer Index Composite (EPIC) questionnaire (ie, “How would you describe the usual quality of your erections during the last 4 weeks?”). Patients who preoperatively responded *None at all* were defined as having no preoperative potency. The EPIC questionnaire is a validated and reliable instrument used to analyze SF and bother after PC treatment, and it has been translated into Japanese.^18^^,^^19^ All patients were preoperatively informed about penile rehabilitation with LIEWST or pharmacotherapy with a PDE5i. In patients who requested penile rehabilitation, those who selected and underwent LIESWT were categorized as the LIEWST group. In contrast, those who underwent pharmacotherapy with a PDE5i were classified as the PDE5i group. Patients who did not request penile rehabilitation were defined as the control group.

**Figure 1:**
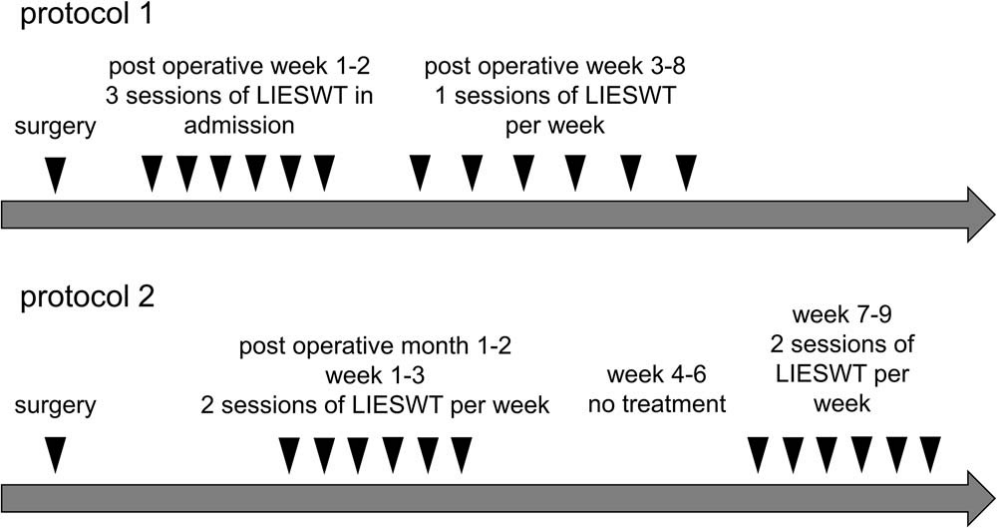
LIESWT treatment protocol in this study. LIESWT, low-intensity extracorporeal shock wave therapy.

LIESWT was performed via a probe attached to a compact electrohydraulic unit with a focused shock wave source (Omnispec ED1000; Medispec) during each session.^13^ The penis was manually stretched, and shock waves were delivered to 5 sites: the ventral side of the distal, mid, and proximal penile shaft and the left and right crura. The treatment duration for each LIESWT session was approximately 20 minutes, and each session consisted of 300 shocks for each treatment site (1500 per session) at an energy density of 0.09 mJ/mm^2^ and a frequency of 120/min. The value of penile tissue exposed to the shock waves at each site was cylindrical. The therapeutical zone was 9 mm in diameter and 135 mm in depth. The tissue volume treated in each pulse was 8.6 cm^3^. Local or systemic analgesia was not required during the procedure. We used 2 LIESWT protocols (Figure 1).^13^ Protocol 1 was modified from that reported by Vardi et al, which was used 3 times a week for 2 weeks during admission and 1 time per week in the outpatient clinic for 6 weeks.^20^ Protocol 2 was the same as that reported by Vardi et al, for which administration comprised 2 treatment sessions per week for 3 weeks after 1 to 2 months postoperatively, which were repeated after a 3-week no-treatment interval. Patients who underwent LIESWT using protocol 1 or 2 were allocated to the LIESWT group. Protocol 2 was followed for patients diagnosed until December 2016, while protocol 1 was followed for patients diagnosed after January 2017. The PDE5i group was prescribed a PDE5i (sildenafil, vardenafil, or tadalafil) and instructed to take it once or twice a week for ≥4 weeks.^21^

### Clinical data collection and outcome

All patients were invited to complete the EPIC and 5-item International Index of Erectile Function (IIEF-5) questionnaires preoperatively and at 3, 6, 12, 24, and 60 months after RARP. Of the EPIC domains, SF was assessed in this study. The IIEF-5 is an abridged version of the 15-item IIEF to evaluate the presence and severity of ED, and it has been translated into Japanese.^22^^,^^23^ Potency and intercourse rate were estimated by answers to a question in the SF domain of the EPIC questionnaire. Potency was defined as “firm enough for masturbation and foreplay only” or “firm enough for intercourse,” while intercourse was described as “Firm enough for intercourse” only. To assess the differences in the recovery of sexual and erectile functions in the LIESWT, PDE5i, and control groups, we compared the serial changes in SF and total IIEF-5 scores and in potency and intercourse rate.

Relevant clinicopathologic data were collected: age, body mass index, initial prostate-specific antigen, prostate volume (measured by transabdominal ultrasonography), pathologic grade according to the International Society of Urological Pathology,^24^ pathologic T stage, risk classification according to the National Comprehensive Cancer Network,^25^ pelvic lymph node dissection (obturator, external iliac, and internal iliac),^26^ nerve-sparing status, serum testosterone level, concomitant disease (diabetes, hypertension, and dyslipidemia), surgical time, and estimated blood loss. Adverse events of LIESWT and PDE5i were also retrospectively reviewed.

### Statistical analysis

All continuous variables are summarized as median and range. Pearson chi-square test was used to compare the distributions of the categorical variables. The differences between the variables with continuous distributions across dichotomous categories were assessed with the Wilcoxon signed rank test. All statistical analyses were conducted with JMP Pro 16.0.0 software (SAS Institute). *P* < .05 denoted statistical significance.

## Results

### Patient characteristics

**Figure 2:**
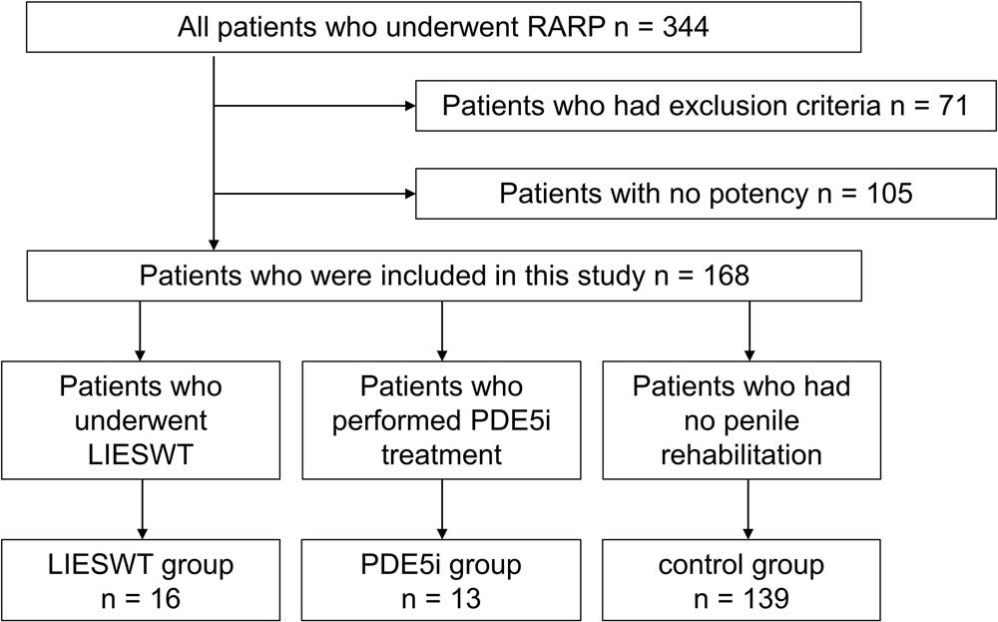
Flowchart for patient selection in this study.

Of the 344 patients, 16 and 13 were assigned to the LIESWT and PDE5i groups, respectively (Figure 2). The remaining 139 patients with no exclusion criteria were included in the control group, while 71 with exclusion criteria and 105 with no preoperative potency were excluded from the study. In the LIESWT group, 6 patients took PDE5i during the follow-up period. Table 1 lists the baseline characteristics of the patients in our cohort: they showed significant differences in age (*P* = .021) and prostate volume (*P* = .017) between the LIESWT and control groups and in age (*P* = .001) and nerve-sparing status (*P* = .027) between the PDE5i and control groups. There was no significant difference between the LIESWT and PDE5i groups in baseline characteristics. During the follow-up period, 1 patient (7.7%) in the PDE5i group had a headache and discontinued the medication. There were no adverse events in the LIESWT group, and all patients completed the sessions.

**Table 1:** Baseline characteristics of all patients.^a^

				***P* value**		
**Variables**	**LIESWT (n = 16)**	**PDE5i (n = 13)**	**Control (n = 139)**	**LIESWT vs Control**	**PDE5i vs Control**	**LIESWT vs PDE5i**
Age, y	63 (52-72)	61 (49-69)	66 (46-75)	.021^b^	.001^b^	.208
Body mass index, kg/m^2^	23.1 (18.5-28.7)	22.9 (18.0-28.7)	23.2 (17.8-26.7)	.892	.984	.809
Initial PSA, ng/mL	5.7 (4.3-18.3)	5.4 (2.8-15.0)	6.6 (2.6-35.6)	.191	.315	.930
Prostate volume, mL	18.5 (11.0-46.0)	22.4 (10.5-48.0)	27.8 (10.8-79.8)	.017^b^	.106	.677
ISUP grade				.735	.221	.472
1, 2	9 (56.3)	9 (69.2)	72 (51.8)			
3, 4, 5	7 (43.8)	4 (30.8)	67 (48.2)			
Pathologic T stage				.997	.277	.379
≤T2a	13 (81.3)	12 (92.3)	113 (81.3)			
≥T3a	3 (18.8)	1 (7.7)	26 (18.7)			
Pelvic lymph node dissection	3 (18.8)	1 (7.7)	23 (16.6)	.826	.364	.379
Nerve-sparing techniques				.333	.027 ^b^	.293
Bilateral/unilateral	8 (50.0)	9 (69.2)	52 (37.4)			
None	8 (50.0)	4 (30.8)	87 (62.6)			
Testosterone ng/mL	3.9 (2.0-9.3)	4.1 (2.4-7.3)	4.2 (1.2-9.2)	.528	.504	.877
Concomitant disease						
Diabetes	1 (6.3)	0 (0.0)	16 (11.5)	.495	.082	.270
Hypertension	2 (12.5)	4 (30.8)	39 (28.1)	.153	.837	.226
Dyslipidemia	3 (18.8)	1 (7.7)	21 (15.1)	.710	.434	.379
Surgical time, min	197 (146-276)	186 (157-417)	189 (119-300)	.506	.461	.809
Estimated blood loss, mL	150 (40-299)	194 (60-1025)	120 (20-715)	.714	.082	.109

Abbreviations: ISUP, International Society of Urological Pathology; LIESWT, low-intensity extracorporeal shock wave therapy; PDE5i, phosphodiesterase type 5 inhibitors; PSA, prostate-specific antigen.

a Data are presented as median (range) or No. (%).

b *P* < .05.

### Serial changes in SF score, IIEF-5 score, and potency of the LIEWST, PDE5i, and control groups after RARP

In this study, the rates of response to the questionnaires for the 168 patients preoperatively and 3, 6, 12, 24, and 60 months postoperatively were 128 (76.2%), 129 (76.8%), 127 (75.6%), 125 (74.4%), 112 (66.7%), and 125 (74.4%), respectively.

**Figure 3:**
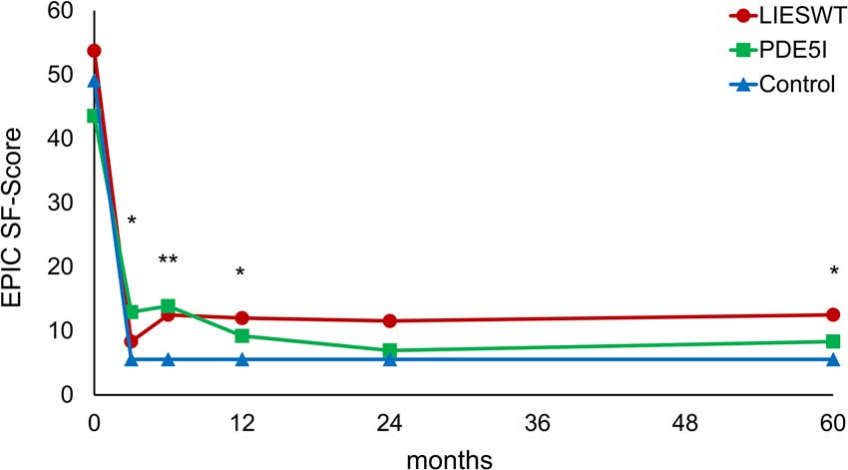
Serial changes in median SF score. LIESWT, low-intensity extracorporeal shock wave therapy; PDE5i, phosphodiesterase type 5 inhibitor; SF, sexual function. ^*^P < .05 (LIESWT vs control or PDE5i vs control). ^*^^*^P < .05 (LIESWT vs control and PDE5i vs control).

The changes in SF score after RARP are shown in Figure 3. The scores of all groups decreased over 3 months after surgery and remained the same. The LIESWT group had significantly higher SF scores than the control group at 6 (*P* = .042), 12 (*P* = .047), and 60 (*P* = .036) months. The PDE5i group had significantly higher SF scores than the control group at 3 (*P* = .011) and 6 (*P* = .038) months. In contrast, no significant difference was observed between the SF scores of the LIESWT and PDE5i groups.

**Figure 4:**
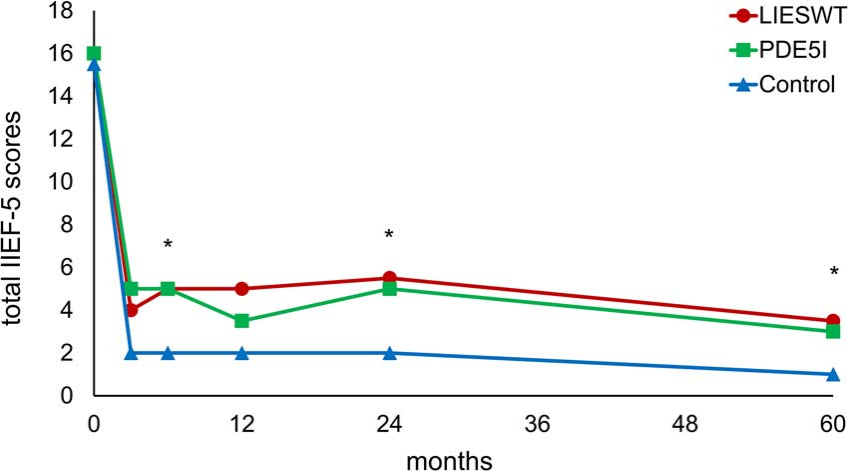
Serial changes in median IIEF-5 score. IIEF-5, 5-item International Index of Erectile Function; LIESWT, low-intensity extracorporeal shock wave therapy; PDE5i, phosphodiesterase type 5 inhibitor. ^*^P < .05 (LIESWT vs control or PDE5i vs control). ^*^^*^P < .05 (LIESWT vs control and PDE5i vs control).

The changes in IIEF-5 scores after RARP are shown in Figure 4. The scores of all groups decreased over 3 months after surgery and remained the same. The LIESWT group had significantly higher IIEF-5 scores than the control group 24 (*P* = .025) and 60 (*P* = .025) months after surgery, whereas the PDE5i group had significantly higher total IIEF-5 scores than the control group at 6 months (*P* = .003). In contrast, no significant difference was observed between the IIEF-5 scores of the LIESWT and PDE5i groups.

**Figure 5:**
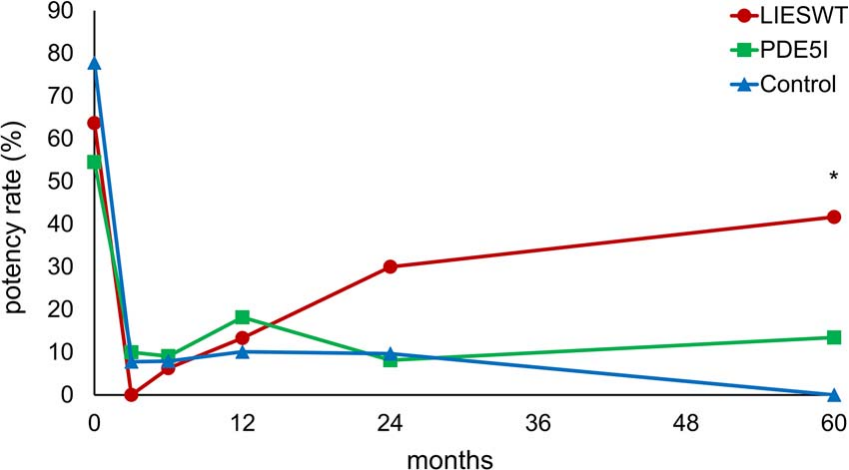
Serial changes in potency. LIESWT, low-intensity extracorporeal shock wave therapy; PDE5i, phosphodiesterase type 5 inhibitor. ^*^P < .05 (LIESWT vs control or PDE5i vs control).

The changes in potency rate after RARP are shown in Figure 5. The potency rate of the LIESWT group decreased over 3 months after surgery and gradually improved thereafter, whereas the other groups maintained almost the same potency rate after surgery. The LIESWT group had a significantly higher potency rate than the control group at 60 months (*P* = .026) after surgery, whereas the PDE5i group showed no significant difference. No significant difference was observed between the potencies of the LIESWT and PDE5i groups.

**Figure 6:**
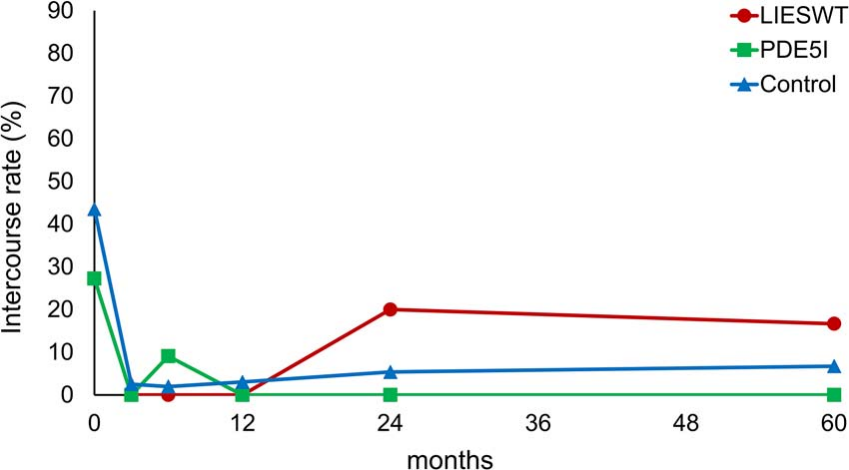
Serial changes in intercourse rate. LIESWT, low-intensity extracorporeal shock wave therapy; PDE5i, phosphodiesterase type 5 inhibitor.

The changes in intercourse rate after RARP are shown in Figure 6. The intercourse rate of the LIESWT group decreased over 3 months after surgery and gradually improved thereafter. In contrast, the other groups maintained almost the same intercourse rate after surgery. However, no significant difference in intercourse rate was observed among the LIESWT, PDE5i, and control groups.

## Discussion

This is the first study to assess the long-term efficacy of LIESWT for penile rehabilitation after RARP, showing that LIESWT improves the sexual and erectile functions of patients with ED after RARP in the long term. Furthermore, LIESWT was not inferior to the PDE5is in improving sexual and erectile functions in the long term after surgery.

In patients who undergo RARP, ED is caused by (1) neuropraxia induced by intraoperative manipulations and penile structural remodeling and (2) smooth muscle apoptosis and fibrosis induced by neurovascular bundle injury.^27^^,^^28^ In preclinical studies, LIESWT enhanced tissue regeneration via its shear stress effect (microtrauma and mechanical stress effects on deep tissue) and increased the expressions of vascular endothelial growth factor and endothelial nitric oxide synthase.^29^ These effects can promote tissue neoangiogenesis and in turn improve blood flow in the penis. LIESWT can prevent neuropraxia and penile structural remodeling after RARP. Previous studies with short-term follow-up showed that LIESWT could improve erectile function in patients with ED after RARP.^12-14^ In this study, we showed that the effect of LIESWT can be sustained over the long term after surgery.

We demonstrated that penile rehabilitation with LIESWT was not inferior to that of the PDE5is in the long term after surgery. PDE5is are standard for penile rehabilitation, and compelling evidence has shown their usefulness as primary treatments for ED after RARP.^5^^,^^30^ However, caution should be exercised with use of a PDE5i because it can induce adverse events such as flushing, dyspepsia, and upper respiratory tract complaints.^30^ A previous study compared the efficacies of LIESWT and PDE5is for penile rehabilitation and reported that either treatment was associated with better recovery of potency than no treatment.^31^ Our results confirmed that the efficacy of LIESWT is sustained over a long period after surgery. Moreover, this study reported no adverse events in the LIESWT group. LIESWT may be a new option for penile rehabilitation in patients who are concerned about adverse events.

LIESWT and PDE5i have different mechanisms for improving erectile function; thus, synergistic effects can be expected. LIESWT enhances tissue regeneration via its shear stress effect and improves blood flow through tissue neoangiogenesis, whereas PDE5is inhibit the breakdown of cyclic guanosine monophosphate in penile tissues, prolonging smooth muscle relaxation and facilitating erection.^32^ Several randomized clinical trials comparing a penile rehabilitation protocol consisting of LIESWT + PDE5i vs PDE5i alone have reported better improvement in erectile function with the combination protocol.^12^^,^^14^ Based on our results, the combination of LIESWT and PDE5i may be effective for ED after RARP over the long term.

Nerve-sparing techniques are necessary for improving the sexual and erectile functions of patients who undergo RARP.^8^ However, ED has persisted as a life-distressing sequel of nerve-sparing RARP for patients. A previous meta-analysis showed that nerve-sparing RARP was associated with a 12-month incidence of ED ranging from 10% to 46%, and ED after nerve-sparing RARP is not rare.^6^ Since LIESWT is capable of restoring the neurovascular bundle injury during nerve-sparing RARP through neoangiogenesis, the combination of nerve-sparing RARP and LIESWT may help preserve sexual and erectile functions. During non–nerve-sparing RARP, not all cavernous nerves are removed from the neurovascular bundle because these nerves cover the rhabdosphincter and perirectum caudal to the apex of the prostate.^33^ Therefore, LIESWT may be useful for penile rehabilitation for ED after non–nerve-sparing RARP.

Our study had several limitations. This is a retrospective single-center study and involved relatively few patients. Furthermore, the selection of this study for penile rehabilitation was not made randomly but by the patient’s choice. The limited sample sizes (16 and 13 patients in the LIESWT and PDE5i groups, respectively) may have led to selection bias. The patient characteristics, including age and nerve-sparing status, were different for the LIESWT, PDE5i, and control groups. In addition, an appropriate protocol of LIESWT for penile rehabilitation after RARP has not been established. Several issues, such as energy density, number of pulses, and treatment duration and interval, need to be investigated to develop an appropriate protocol for LIESWT during penile rehabilitation. Another problem in this study was that using different LIESWT protocols could be an additional bias factor. To appropriately test the effectiveness of LIESWT in recovering erectile function following RP, a randomized controlled trial is needed that includes a large cohort and compares 2 groups: shockwave therapy and sham (same machine but without shockwave emission). In this study, the PDE5i group was prescribed PDE5i once or twice a week. However, a recent study showed that regular high doses of PDE5i could significantly increase the rate of erectile function recovery after RP.^34^ In comparing the effects of LIESWT and PDE5i, the high dose of the PDE5i protocol must also be reconsidered.

Despite these limitations, the results of our pilot study are valuable: they support the evidence of LIESWT for penile rehabilitation after RARP, and they are based on the first study to assess the long-term efficacy of LIESWT. We plan to confirm our results in a future study with a larger sample and long-term follow-up.

## Conclusion

LIESWT can improve sexual and erectile function in patients with ED after RARP, and the efficacy of LIESWT can be maintained in the long term after RARP. Furthermore, the effect of LIESWT on ED after RARP was not inferior to that of PDE5i, which means that LIESWT may be a new option for penile rehabilitation.

## Funding

None declared.

*Conflicts of interest:* None declared.
